# Mental wellbeing of higher education students in challenging times

**DOI:** 10.3389/fpubh.2024.1368443

**Published:** 2025-01-07

**Authors:** Magdalena Anna Lazarewicz, Unni Karin Moksnes, Randi Johansen Reidunsdatter, Dorota Wlodarczyk

**Affiliations:** ^1^Department of Health Psychology, Medical University of Warsaw, Warsaw, Poland; ^2^Department of Public Health and Nursing, Norwegian University of Science and Technology, Trondheim, Norway; ^3^Department of Circulation and Medical Imaging, Norwegian University of Science and Technology, Trondheim, Norway

**Keywords:** university students, wellbeing, global challenges, pandemic, cognitive appraisal, personal resources

## Abstract

**Objective:**

Student age and starting higher education require adaptation to a new physical and psychosocial environment, making the time of studies a highly sensitive period. Current and future generations of students are also likely to face additional global stressors, which potentially exacerbate their mental wellbeing. The aim of the study was to investigate how higher education students' appraisal of the COVID-19 pandemic situation and of their personal resources predict mental wellbeing (anxiety and curiosity).

**Methods:**

The study used cross-sectional data collected from 3,727 higher education students in an online survey conducted during the first wave of the COVID-19 pandemic.

**Results:**

After controlling for a number of pandemic-related factors, all considered aspects of the pandemic situation appraisal and resources appraisal were significant predictors of anxiety and/or curiosity. The most important predictors of anxiety were sense of control, information stress, pandemic interest, and self-efficacy, and the most important predictors of curiosity were self-efficacy, health promoting behaviors, and sense of control. Tested models explained 61% of variance of anxiety and 36% of variance of curiosity.

**Conclusion:**

Appraisal of own personal resources seems critical for both indicators of mental wellbeing. These results may significantly contribute to sufficient planning of mental-wellbeing oriented interventions for young adults in Higher Education.

## 1 Introduction

Each stage of development presents its own challenges and opportunities. For young people, these are primarily associated with adolescence, often considered the most critical developmental stage, and young adulthood, particularly in the context of higher education (HE). Admission to college or university requires adaptation to a new physical and psychosocial environment, making the time of studies a highly sensitive period. Many HE students leave their family homes, which can be associated with feelings of increased independence and autonomy. However, this transition may also involve challenges such as adjusting to new responsibilities, reduced access to familiar social support, and feelings of homesickness or isolation (1, 2). The extent to which these experiences are felt can vary depending on cultural and individual factors and may be particularly pronounced in collectivist societies.

HE students develop and mature academically but also need to continuously adjust and cope with various academic demands, the competitive learning environment and the fear of failure. They may also experience lack of time for self-care or social life with family and friends (3). At the same time, student age itself is associated with many developmental challenges—it is a time of shaping one's own identity and trying to establish close relationships (4). The above may result in high levels of psychological problems often characterized by high comorbidity (3, 5, 6).

Besides common challenges of student age and studying in a HE institution, current and future generations of students are likely to face additional stressors such as global climate changes, natural disasters, military conflicts or new pandemics. An example of such global challenge was the COVID-19 pandemic, which potentially exacerbated HE students' mental health.

Although some studies show no significant increase in anxiety and depression levels among young adults in the period before and during the first lockdown (7), most studies report that students' mental health deteriorated during the early stage of the COVID-19 pandemic (8–13). At the beginning of the COVID-19 pandemic, young people aged 18 to 24 reacted with the highest intensity of depression and anxiety (14). The student population also recorded the strongest increase in loneliness compared to the pre-pandemic period, from 9% to 35% (15). This moved young people from the least lonely age group in the past to the loneliest group at the beginning of the pandemic. In Poland, between years 2019 and 2021, the highest increase in the number of suicides was recorded in the 19 to 24 age group and teenagers (16). Over 70% of students indicated a deterioration in their own mental health since the outbreak of the pandemic, with the percentage of students reporting mental problems gradually decreasing as pandemic-related restrictions were loosened (17). The pandemic-related social restrictions caused a severe reduction in wellbeing in almost 75% of students (18). Social restrictions, lack of interactions and emotional support as well as physical isolation have been linked to students' negative mental health trajectories (19) and change in health behavior (20). At the same time, it may be expected that social networking and social support would have a positive impact on students' wellbeing and their sense of belonging. Unsurprisingly, female students appeared to have poorer mental health than male students after controlling for various levels of social inclusion and COVID-19-related stressors (19, 21).

The outbreak of the pandemic was an additional stressful situation with the potential to interfere with one's daily life, by disturbing the progress of one's action or impeding and/or jeopardizing the satisfaction of needs or realization of tasks (22). According to Lazarus and Folkman's (23) well-established transactional model of stress, “*psychological stress is a particular relationship between the person and the environment that is appraised by the person as taxing or exceeding his or her resources and endangering his or her* wellbeing” (p. 19). The key focus here is on the actual processes of perception and response selection, which link particular stressors to particular outcomes (24). Hence, not the situation itself but the individual interpretation of how (potentially) harmful a particular situation is (primary appraisal) and the evaluation of whether a person possesses the resources to successfully face the demands of this situation (secondary appraisal) are crucial for the stress process and the outcome of the stress process on health.

Different aspects of the appraisal of the situation may be significant for students' wellbeing. One of them is general *pandemic interest*—the level of attention, focusing vs. intentionally or subconsciously avoiding pandemic-related information. People seek information for informed decision-making. However, information from multiple sources can lead to information overload, which then creates anxiety, stress, fatigue, exhaustion, and in turn may result in information avoidance (25–27). Among different information sources, exposure to social media has a significant relationship with information overload as well as information anxiety (27). Additionally, one of the challenging factors accompanying the COVID-19 pandemic, especially in its early stages, was the wave of dis- and misinformation, a phenomenon known as a “COVID-19 infodemic” (28). We were inundated with information of varying levels of credibility, often contradictory, by specific scientific facts, conspiracy theories, and “fake news,” which made it challenging to assess how harmful the situation was. Ironically, studies suggest that attention given to COVID-19 conspiracy theories may inflate the problem: describing or explaining the existence of COVID-19 conspiracies may increase support for them and undermine knowledge about and the willingness to engage in COVID-19 mitigation (29). Thus, *information stress* caused by the infodemic may be seen as another indicator of the situation appraisal. Research has also shown that the *perception of the pandemic-related health risk* has a significant impact on how people manage their mental wellbeing (30) and whether they protect themselves and practice preventive behaviors (31, 32). During the pandemic, high-risk perception intensified feelings of fear and anxiety among the general public (33) and expanded involvement in seeking knowledge about this global threat (34).

The individual appraisal of one's resources to successfully face the demands of a situation, such as self-efficacy and sense of control, is critical for mental wellbeing in the context of such global challenges as the COVID-19 pandemic. *Self-efficacy*, an individual's belief in their capacity to execute behaviors necessary to successfully complete tasks and achieve goals (35, 36), is a well-studied personal resource, which, among others, is found to be a significant predictor of posttraumatic recovery, e.g., among collective trauma survivors (37). *Sense of control*, the belief that you can and do master, control, and shape your own life, is partially dependent on the characteristics of the given situation. New, unclear, uncertain, and unpredictable situations—such as the early stage of the COVID-19 pandemic—may lead to a decrease in a personal sense of control. People who experience loss of control and psychological burden are prone to dysfunctional coping strategies that could negatively impact their mental and physical health (38, 39).

Therefore, the appraisal processes and its outcome—the assessment of the demand–resources balance—are critical for assessing the level of HE students adaptation to various current demands. It also seems important to not only investigate the pathological aspects of psychological distress (e.g., levels of anxiety, anger, and depression) but also the more positive dimensions of mental wellbeing e.g., levels of curiosity considered as a positive emotional vital sign (40).

The aim of the study was to investigate how the appraisal of the situation and own resources relate to HE students' mental wellbeing in the situation of cumulative stress. We formulated the following research questions: (1) How were the three aspects of the primary appraisal (information stress, perceived risk, pandemic interest) related to HE students mental wellbeing (anxiety and curiosity)? (2) How were the two aspects of the secondary appraisal (self-efficacy and sense of control) related to HE students mental wellbeing (anxiety and curiosity)? (3) How were other factors (demographic, contextual, social and health behaviors) related to HE students mental wellbeing (anxiety and curiosity)?

## 2 Methodology

### 2.1 Procedure

The study is based on cross-sectional survey. Data were collected online during the first wave of the COVID-19 pandemic in Poland, between April 23 and June 30, 2020 (till the end of the academic year). Requests to forward a study invitation to HE students were sent by email to the authorities (rectors and deans of all faculties) of 30 out of 99 randomly selected public Polish HE institutions, student governments' representatives of these institutions, and to 15 Polish student associations and organizations. We used Research Randomizer (https://www.randomizer.org/) to generate a random sample of HE institutions, proportional to different disciplines (e.g., humanistic universities, medical universities, technical universities/polytechnics, naval and military academies, economic schools, agricultural academies, artistic schools, and HEI of physical education). Respondents were also reached via social media (Facebook and Instagram) on various student groups and forums. We used snowball sampling, encouraging students to share the invitation to the study with their acquaintances.

The survey was prepared using Google Forms, and the participants responded to it anonymously. Responding to the survey was taken as consent to participate in the study. Participants were encouraged to share their email address in case of further contact.

### 2.2 Participants

**Figure 1:**
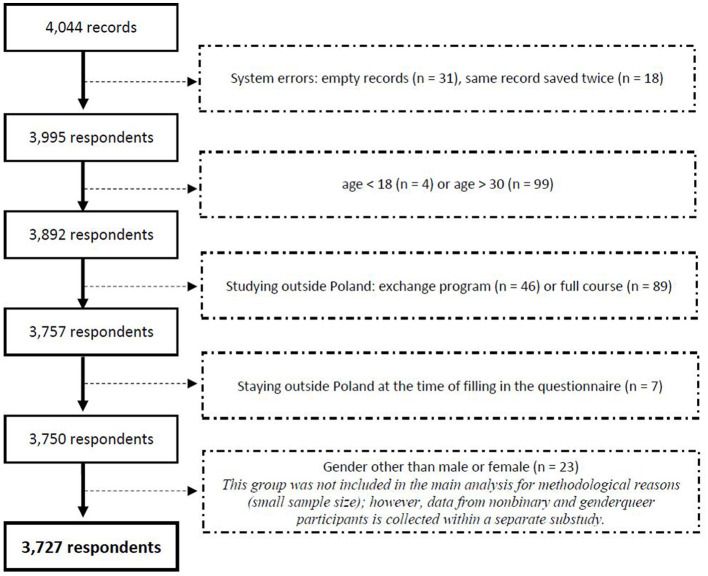
Data cleaning process.

The study population consists of Polish bachelor, master and PhD students of Polish HEI. Additionally, we applied the following inclusion criteria: (a) age between 18 and 30, and (b) studying and staying in Poland during the spring semester of the data collection. Data were collected from 3,995 participants, and 3,727 of them met the study conditions. Figure 1 illustrates the number of excluded respondents at each part of the data cleaning procedure.

### 2.3 Measures

#### 2.3.1 Mental wellbeing, personal resources and situation appraisal

Due to a general overload of students with online activities after the onset of the pandemic, a study specific questionnaire was developed, largely based on already existing validated scales. The questionnaire included a number of two-item scales measuring i.a. anxiety, curiosity, self-efficacy, sense of control and information stress. All items were formulated in the same pattern starting with an unfinished statement: *Referring to the SARS-CoV-2 coronavirus pandemic and taking into account all areas of your life, to what extent during the last week you…*. Respondents were asked to rate their experience or feeling on a 5-point Likert scale from 1—*not at all* to 5—*very much*. Results on the scales were expressed as mean scores from the two given items, and higher scores indicated higher levels of the measured variables. All scales are presented in Supplementary Material S1a. The Cronbach's alphas for the scales used in the study ranged from 0.78 to 0.86. To assess students' mental wellbeing, we used the anxiety (e.g., ...*you have been worried about what the next days will bring*) and curiosity (e.g., ...*you were full of enthusiasm*) scales. To assess personal resources (secondary appraisal), we used the self-efficacy scale (e.g., ...*you were confident that you could deal efficiently with unexpected events*) and the sense of control scale (e.g., ...*you have had a feeling that you have no influence on what is happening around you*). To assess the COVID-19 pandemic situation's appraisal (primary appraisal), we used the information stress scale (e.g., *...have you felt overwhelmed with information and have you had difficulty controlling it*) and two additional indicators: (a) Students' perceived own risk of getting infected or dying due to COVID-19 measured by a perceived own risk index calculated as a mean of estimations of *the risk of getting infected with SARS-CoV-2 coronavirus* and *the risk of coronavirus infection being fatal* in the student's individual case. The estimations were given in percentages; (b) Pandemic interest measured by a single item (*In general, how much are you interested in the coronavirus SARS-CoV-2 pandemic*?) with answers on a 5-point Likert scale from 1—very little to 5—very much. The above measures of the primary appraisal of the COVID-19 situation were treated as independent indicators.

#### 2.3.2 Students' experience of the COVID-19 pandemic

Students' experience of the COVID-19 pandemic was estimated by the following contextual factors: (a) current situation in the country as perceived by students, measured by a 12-item COVID-19 Situation Index (e.g., *Due to the COVID-19 pandemic: we experience shortages of some products, e.g., toilet paper, soap, flour; all mass events are canceled; universities are closed; borders are closed; field hospitals are open*) where answers *neither now nor in the past* and *in the past but not anymore* were classified as 0 and *yes, at the moment* was classified as 1. The index was expressed as a mean score from the 12 items (see Supplementary material S1b). A possible sum score of the index was between 0 and 1, with a higher score indicating more changes and limitations due to the COVID-19 pandemic; (b) the subjective level of “life's normality,” measured by a single item: *In the country where I currently live, we lead normal lives despite the COVID-19 pandemic*, with a 5-point response format from 1—*definitely not* to 5—*definitely yes;* (c) students' personal experience with COVID-19, measured by two separate dichotomized items: *Do you personally know someone who has been confirmed as having SARS-CoV-2 coronavirus infection?* and *Have you been quarantined*? The response format was 0—no and 1—yes.

#### 2.3.3 Social context

To assess social factors, we asked the students to estimate *with whom they stayed in touch with on a daily basis (e.g., by phone, email or other remote technologies) during the SARS-CoV-2 coronavirus outbreak?* Students could answer on seven categories (colleagues, close friends, significant others (if applicable), close family, extended family, neighbors, lecturers) with response options from 1—*definitely not* to 5—*definitely yes*. We created two indexes from these answers: (1) contact intensity with peers, expressed as a mean score from the intensity of contact with colleagues, close friends, and significant others, and (2) contact intensity with family, expressed as a mean score from the intensity of contact with close and extended family. The remaining two answers (neighbors and lecturers) were analyzed as single items.

#### 2.3.4 Health behaviors

Students' health behaviors in the context of the COVID-19 pandemic were measured by two checklists: (a) a 15-item preventive behaviors checklist (e.g., *I avoid crowded places, I wear a mask in public places, I wash my hands with soap for min. 30 seconds*) and (b) a 6-item health-promoting behaviors checklist [e.g., *I sleep at night for at least 7 hours, I eat about 5 servings (400 g) of vegetables daily, I take vitamin D*]. Both checklists had a response format from 1—not at all to 7—all the time. The checklists' scores were expressed as mean scores from the 15 and 6 items, respectively (see Supplementary material S1c). Higher scores indicated higher levels of preventive and promoting behaviors.

#### 2.3.5 Demographic information

Participants were also asked to report demographic information (age, gender, current place of residency, living arrangements) and study-related information (field of study, level and year of studies).

### 2.4 Statistical analysis

First, initial descriptive analyses and examination of intercorrelations between demographic factors, contextual factors, social factors, health behaviors, primary and secondary appraisal, and wellbeing were performed. Next, multivariate linear regression models were designed to investigate potentially significant predictors of student wellbeing. The assumptions of linearity, normally distributed errors, uncorrelated errors, no perfect multicollinearity and homoscedasticity were checked and met. Regression analyses were conducted separately for anxiety and curiosity as dependent (outcome) variables. Predictors were entered to regression analyses in the following order:

– Step 1. Demographics: age, gender.
– Step 2. Contextual factors: COVID-19 situation index, normal life, know infected people, quarantine.
– Step 3. Social factors: contact intensity with peers, family, lecturers and neighbors.
– Step 4. Health behaviors: preventive and health-promoting behaviors.
– Step 5. COVID-19 pandemic situation appraisal (primary appraisal): perceived own risk, information stress, pandemic interest.
– Step 6. Resources appraisal (secondary appraisal): self-efficacy, sense of control.

All statistical analyses were conducted using the Statistical Package for Social Sciences Statistics (IBM, Armonk, NY, United States, version 27.0).

## 3 Results

### 3.1 Sample characteristics and variable descriptions

Demographic characteristic together with mean scores and standard deviations of social factors, health behaviors, pandemic situation, and resources appraisal indicators and students' wellbeing are presented in Table 1.

**Table 1:** Characteristics of the student sample aged 18–30 years (*n* = 3,727): means, standard deviations or frequencies and proportions.

	***M* (SD)**	***n* (%)**		***M* (SD)**
**Demographic characteristics**			**Social factors**	
Age (18–30)	21.96 (1.98)		Contact intensity (1–5)^d^	
Gender^a^			Peers	3.73 (0.94)
Female		2,772 (74.4)	Family	2.21 (0.58)
Male		955 (25.6)	Lecturers	3.11 (1.29)
Field of study			Neighbors	1.76 (1.07)
Medical		151 (41.9)		
Arts and humanities		849 (22.7)	**Health behaviors**	
Sciences		1,004 (26.9)	Preventive (1–7)^e^	5.27 (1.05)
Other		315 (8.5)	Health-promoting (1–7)^e^	4.07 (1.12)
Level of studies				
Bachelors		1,797 (48.2)	**Pandemic situation appraisal**	
Master's or 6-year medicine		1,898 (50.9)	Perceived own risk (0–100%)	
PhD		32 (0.9)	COVID-19 infection	33.5 (24.9)
Current place of residence			Death due to COVID-19	14.4 (20.0)
Countryside		1,077 (28.9)	Mean infection/death	23.9 (18.2)
City up to 50,000		655 (17.6)	Information stress (2–10)^f^	6.87 (2.59)
City 50,001–100,000		319 (8.6)	Pandemic interest (1–5)^g^	3.39 (0.98)
City 100,001–500,000		563 (15.1)		
City over 500,001		1,113 (29.9)	**Resources appraisal**	
Living arrangements			Self-efficacy (2–10)^f^	5.87 (2.23)
Dormitory		25 (0.7)	Sense of control (2–10)^f^	4.83 (2.54)
Alone in private accommodation		156 (4.2)		
With flatmate(s)		254 (6.8)	**Wellbeing**	
With family		2,810 (75.4)	Anxiety (2–10)^f^	7.07 (2.40)
With a spouse or partner		381 (10.2)	Curiosity (2–10)^f^	4.66 (2.04)
with partner and children		37 (1.0)		
Other		64 (1.7)		
**Contextual factors**				
COVID-19 situation index (0–1)^b^	0.51 (0.19)			
Normal life (1–5)^c^	2.36 (1.03)			
Knows infected people				
Yes		623 (16.7)		
No or I don't know		3,104 (83.3)		
Loved one died due to COVID-19				
Yes		21 (0.6)		
No		3,668 (98.4)		
I don't know		38 (1.0)		
Quarantined				
No		3,546 (95.1)		
Yes		181 (4.9)		
How many days?	13.1 (4.91)		

M, mean; SD, standard deviation.

^a^Data from nonbinary and genderqueer participants (n = 23) were not included in the analysis in this study for methodological reasons (small sample size); it is collected within a separate substudy.

^b^Higher score indicates more changes and limitations due to the COVID-19 pandemic.

^c^Higher score means more “normal” life.

^d^Higher score indicates more intensive contact.

^e^Higher scores mean higher levels of preventive and health-promoting behaviors.

^f^Higher scores mean higher levels of information stress, self-efficacy, sense of control, anxiety and curiosity.

^g^Higher score indicates higher general interest in the pandemic.

The mean age of the students was 22.0 years. Almost three quarters of them were female. The biggest group of students was from the medical field (42%), followed by students in the sciences (27%) and arts and humanities (23%). Half of the participants were studying for a bachelor's and half for a master's degree. During the time of the data collection, approximately one-third of the students lived in the countryside, another one-third in large cities with over 500,000 citizens, and the last one-third in smaller towns and cities, with 50,000–500,000 citizens. Most students lived with their family: parents, siblings, and/or grandparents (75%) or with a spouse/partner (11%). In the opinion of 58% of the students, at the time of the data collection people in Poland did not or definitely did not lead normal lives due to the COVID-19 pandemic. Their immediate experience with COVID-19 was limited: 5% of the students were quarantined, 17% of them knew someone who had been infected, and <1% lost a loved one due to the pandemic.

### 3.2 Association between predictors and students' mental wellbeing

Intercorrelations between the multiple regression variables are reported in Table 2, and the regression statistics from the final step only are presented in Table 3. Detailed results pertaining to the remaining steps are presented in Supplementary Table 1.

**Table 2:** Correlations between demographic factors, contextual factors, social factors, health behaviors, COVID-19 pandemic situation appraisal, resources appraisal, and student wellbeing (*n* = 3,727).

	**Wellbeing**		**Demographic**		**Contextual**				**Social**				**HB**		**Pandemic situation appraisal**			**Resources appraisal**	
	**Anxiety**	**Curiosity**	**Age**	**Gender**	**COVID-19 situation index**	**Normal life**	**Know infected people**	**Quarantine**	**Contact family**	**Contact peers**	**Contact neighbors**	**Contact lecturers**	**HB_Prev**	**HB_Promo**	**Perceivedown risk**	**Information stress**	**Pandemic Interest**	**Self-Efficacy**	**Sense of Control**
Anxiety	1	−0.28^**^	−0.01	−0.24^**^	0.10^**^	−0.15^**^	0.07^**^	0.01	0.02	0.02	−0.05^**^	0.05^**^	0.24^**^	−0.08^**^	0.22^**^	0.66^**^	0.25^**^	−0.43^**^	−0.73^**^
Curiosity		1	0.04^**^	0.06^**^	0.03^*^	0.04^**^	0.02	−0.01	0.13^**^	0.10^**^	0.10^**^	0.06^**^	0.01	0.28^**^	−0.04^**^	−0.25^**^	0.02	0.55^**^	0.37^**^
Age			1	0.04^*^	−0.04^**^	−0.05^**^	0.06^**^	0.02	0.04^**^	−0.01	−0.001	−0.07^**^	0.02	0.01	0.06^**^	−0.06^**^	0.05^**^	0.05^**^	0.04^**^
Gender				1	−0.02	0.03^*^	−0.05^**^	−0.03	−0.08^**^	−0.08^**^	0.03	−0.07^**^	−0.19^**^	−0.11^**^	−0.13^**^	−0.18^**^	−0.06^**^	0.17^**^	0.23^**^
COVID-19 situation index					1	−0.39^**^	0.001	−0.03^*^	−0.02	0.002	−0.05^**^	−0.05^**^	0.25^**^	0.11^**^	−0.002	0.07^**^	0.08^**^	−0.02	−0.10^**^
Normal life						1	0.03	0.01	−0.001	0.03	0.07^**^	0.02	−0.17^**^	−0.04^**^	0.02	−0.11^**^	−0.10^**^	0.07^**^	0.15^**^
Know infected people							1	0.13^**^	0.04^*^	0.06^**^	0.05^**^	0.02	0.04^**^	0.02	0.11^**^	0.04^*^	0.05^**^	0.01	−0.04^**^
Quarantine								1	0.03^*^	0.04^**^	0.04^*^	0.02	0.004	−0.01	0.06^**^	0.02	0.02	−0.01	−0.03
Contact family									1	0.27^**^	0.32^**^	0.08^**^	0.06^**^	0.14^**^	0.02	0.05^**^	0.07^**^	0.11^**^	0.05^**^
Contact peers										1	0.13^**^	0.17^**^	0.04^**^	0.09^**^	0.04^*^	0.07^**^	0.05^**^	0.10^**^	0.02
Contact neighbors											1	0.10^**^	−0.06^**^	0.05^**^	0.05^**^	0.01	−0.02	0.10^**^	0.07^**^
Contact lecturers												1	0.06^**^	0.02	0.04^*^	0.07^**^	0.06^**^	0.03	−0.02
HB_Prev													1	0.18^**^	0.17^**^	0.13^**^	0.38^**^	−0.08^**^	−0.13^**^
HB_Promo														1	−0.04^**^	−0.09^**^	0.07^**^	0.21^**^	0.14^**^
Perceived own risk															1	0.18^**^	0.17^**^	−0.13^**^	−0.18^**^
Information stress																1	0.13^**^	−0.34^**^	−0.71^**^
Pandemic interest																	1	−0.05^**^	−0.14^**^
Self-efficacy																		1	0.46^**^
Sense of control																			1

Dichotomous variables were coded as follows: gender: female = 0, male = 1; know infected people: no = 0, yes = 1; been quarantined: never = 0, at least once = 1; providing help: never = 0, at least once = 1.

HB_Prev, preventive health behaviors; HB_Promo, health-promoting behaviors.

^*^p < 0.05.

^**^p < 0.01.

**Table 3:** Predicting anxiety and curiosity from demographic factors, contextual factors, social factors, health behaviors and COVID-19 pandemic situation and resources appraisal—multivariate hierarchical linear regression estimates, *N* = 3,727 (Bs from step 6 shown).

**Model**	**Anxiety**			**Curiosity**		
	***B***	**SE** ***B***	**Beta**	***B***	**SE** ***B***	**Beta**
Step 6 (Constant)	5.82	0.39		−1.05	0.42	
**Demographic factors**						
Age	0.03	0.01	0.02^*^	0.01	0.01	0.01
Gender	−0.20	0.06	−0.04^***^	−0.11	0.07	−0.02
**Contextual factors**						
COVID-19 situation index	0.003	0.15	0.000	0.44	0.16	0.04^**^
Normal life	−0.05	0.03	−0.02	0.02	0.03	0.01
Know infected people	0.16	0.07	0.03^*^	0.04	0.07	0.01
Quarantine	−0.18	0.12	−0.02	−0.03	0.13	−0.003
**Social factors**						
Contact family	0.06	0.05	0.02	0.13	0.05	0.04^**^
Contact peers	0.02	0.03	0.01	0.02	0.03	0.01
Contact neighbors	−0.02	0.03	−0.01	0.04	0.03	0.02
Contact lecturers	0.03	0.02	0.01	0.06	0.02	0.04^**^
**Health behaviors**						
HB_Prev	0.18	0.03	0.08^***^	−0.003	0.03	−0.001
HB_Promo	−0.01	0.02	−0.003	0.28	0.03	0.15^***^
**Pandemic situation appraisal**						
Perceived own risk	0.01	0.001	0.04^***^	0.004	0.002	0.03^*^
Information stress	0.23	0.01	0.25^***^	0.02	0.02	0.03
Pandemic interest	0.25	0.03	0.10^***^	0.09	0.03	0.04^**^
**Resources appraisal**						
Self-efficacy	−0.11	0.01	−0.11^***^	0.40	0.01	0.44^***^
Sense of control	−0.43	0.02	−0.46^***^	0.15	0.02	0.18^***^

Dichotomous variables were coded as follows: gender: female = 0, male = 1; know infected people: no = 0, yes = 1; been quarantined: never = 0, at least once = 1; providing help: never = 0, at least once = 1.

HB_Prev, preventive health behaviors; HB_Promo, health-promoting behaviors.

The entire group of predictors significantly predicted anxiety F (17, 3709) = 350.1, p < 0.001, adjusted R^2^ = 0.61 and curiosity F(17, 3709) = 122.1, p < 0.001, adjusted R^2^ = 0.36.

Delta R^2^ statistics for anxiety: R^2^ = 0.06 for Step 1, ΔR^2^ = 0.03 for Step 2 (p < 0.001), ΔR^2^ = 0.003 for Step 3 (p = 0.025), ΔR^2^ = 0.05 for Step 4 (p < 0.001), ΔR^2^ = 0.36 for Step 5 (p = 0.001), ΔR^2^ = 0.12 for Step 6 (p < 0.001).

Delta R^2^ statistics for curiosity: R^2^ = 0.005 for Step 1, ΔR^2^ = 0.005 for Step 2 (p = 0.001), ΔR^2^ = 0.03 for Step 3 (p < 0.001), ΔR^2^ = 0.07 for Step 4 (p < 0.001), ΔR2 = 0.05 for Step 5 (p < 0.001), ΔR^2^ = 0.20 for Step 6 (p < 0.001).

^*^p < 0.05.

^**^p < 0.01.

^***^p < 0.001.

In the case of anxiety, the whole regression model explained 61% of its variance. *Demographic factors* contributed significantly to the regression model, *F*_(2,3,724)_ = 108.66, *p* < 0.001 and accounted for 5.5% of the variation in anxiety. Introducing *contextual factors* to the regression model explained an additional 2.6% of the variation in anxiety, and this change in *R*^2^ was significant, *F*_(6,3,720)_ = 55.08, *p* < 0.001. Adding *social factors* explained an additional 0.3% of the variation in anxiety, and this change in *R*^2^ was significant, *F*_(10,3,716)_ = 34.23, *p* < 0.001. The introduction of *health behaviors* to the regression model explained an additional 4.8% of the variation in anxiety, and this change in *R*^2^ was also significant, *F*_(12,3,714)_ = 47.15, *p* < 0.001. Finally, the addition of the *pandemic appraisal* in steps five (pandemic situation appraisal) and six (resources appraisal) explained an additional 36.3% and 12.1% of variation in anxiety, respectively. These changes in R^2^ squares were also significant, with *F*_(15, 3, 711)_ = 242.87, *p* < 0.001 for step five and *F*_(17,3,709)_ = 350.12, *p* < 0.001 for step six. The most important predictors of anxiety were sense of control (which uniquely explained 8% of the variation in anxiety), information stress (3%), pandemic interest (0.9%), and self-efficacy (0.8%), followed by preventive health behaviors (0.5%), perceived own risk of getting infected or dying due to COVID-19 (0.1%), gender (0.1%), knowing infected people (0.06%), and age (0.05%). Together, the above-mentioned variables accounted for 48% of the variance in anxiety.

In the case of curiosity, the whole regression model explained 36% of its variance. In step one, *demographic factors* contributed significantly to the regression model, *F*_(2,3,724)_ = 10.18, *p* < 0.001) and accounted for 0.5% of the variation in curiosity. Introducing *contextual factors* explained an additional 0.5% of variation in curiosity, and this change in *R*^2^ was also significant, *F*_(6,3,720)_ = 6.48, *p* < 0.001. Adding *social factors* to the regression model explained an additional 2.8% of the variation in curiosity, and this change in *R*^2^ was significant, *F*_(10,3,716)_ = 14.68, *p* < 0.001. Introducing *health behaviors* to the regression model explained an additional 7.4% of the variation in curiosity, and this change in *R*^2^ was also significant, *F*_(12,3,714)_ = 38.92, *p* < 0.001. The addition of the pandemic appraisal at steps five (pandemic situation appraisal) and six (resources appraisal) explained an additional 46% and 19.9% of variation in curiosity, respectively, and the changes in *R*^2^ squares were also significant, with *F*_(15,3,711)_ = 46.49, *p* < 0.001 for step five and *F*_(17,3,709)_ = 121.11, *p* < 0.001 for step six. The most important predictors of curiosity were self-efficacy (which uniquely explained 14.5% of the variation in curiosity), health-promoting behaviors (2%), and sense of control (1.4%), followed by pandemic interest (0.1%), COVID-19 situation index (0.1%), intensity of contact with family (0.1%) and lecturers (0.1%), and perceived own risk of getting infected or dying due to COVID-19 (0.1%). Together the above variables accounted for 17.3% of the variance in curiosity.

## 4 Discussion

The study focuses on how selected aspects of primary and secondary appraisals predicted students' mental wellbeing in a highly demanding situation—the time of the first wave of the COVID-19 pandemic. The study was conducted in the time of the strongest governmental restrictions and the beginning of their gradual lifting. During this period, the 7-day rolling average of daily new confirmed COVID-19 cases in Poland ranged from 82 on April 23 to 267 on June 30, 2020, peaking at 453 on June 12. The 7-day rolling average of daily new confirmed COVID-19 deaths in Poland was at 20 and 13, respectively, peaking at 28 on April 29. The government stringency index (a composite measure based on nine response indicators including school closures, workplace closure and travel bans, rescaled to a value from 0 to 100, with 100 being the strictest), ranged from 83 on April 23 to 51 on June 30 (41). All university classes took place online; however, many governmental restrictions were gradually lifted. First, shopping centers, libraries, museums, art galleries, hotels, medical rehabilitation facilities, nurseries and kindergartens were opened on May 4, 2020. Then, the operation of restaurants, bars, cafes, and hairdressing and beauty salons was restored on May 18. The activity of cultural institutions, i.e., cinemas, theatres, swimming pools and saunas, fitness clubs, and playgrounds was resumed on June 6 (42). More than half of participating students affirmed that during that time people in Poland did not lead normal lives. After controlling for a number of pandemic-related factors, all considered aspects of the COVID-19 pandemic situation appraisal (perceived own risk, information stress, pandemic interest) and resources appraisal (self-efficacy, sense of control) were significant predictors of anxiety and/or curiosity. Both tested models explained a substantial portion of variance of the outcome variables.

### 4.1 Situation appraisal and HE students wellbeing

Out of three analyzed aspects of the pandemic situation appraisal (primary appraisal), information stress had the strongest association with anxiety but was insignificant in association with curiosity. As in previous studies (26, 27) being overwhelmed with information broadcasted and reproduced by the mass and social media was associated with higher anxiety. In response to a new and unknown health threat, seeking information can be one of the most adaptive reactions, and higher news consumption is typical. Thus, proper interest in the situation and adequate evaluation of infection risks are key for effective coping. This is probably why pandemic interest was related positively not only to anxiety but also to curiosity. It would be in line with the general knowledge that some level of anxiety is beneficial in adaptation to a new and changing environment. However, another study carried out among students at the beginning of the COVID-19 pandemic confirmed a fairly general occurrence of unrealistic optimism, slightly stronger in men than in women (43). In the situation of an imminent coronavirus pandemic, students perceived themselves as having less risk of the disease than others in the same risk category. It is believed that the magnitude of this effect depends on the level of perceived controllability of the situation, as if the event is accompanied with a lack of controllability (like an earthquake), unrealistic pessimism would be more likely (44). Unrealistic optimism, through overestimation of one's immunity to adversity, may—similarly to information avoidance (27)—provide some short-term psychological benefits, but it could have a negative impact on health and health behaviors (45). It happened rather quickly that alongside the COVID-19 pandemic, the problem of the information epidemic or infodemic appeared. Information about COVID-19 was often contradictory and focused on the threat, thus raising the level of fear. At the same time, especially at the beginning of the pandemic, it gave little possibility of effective action with a chance to overcome the problem. Thus, high interest in pandemic-related information might instead have resulted in increasing anxiety. The results give answer to our first research question on the relation between HE students appraisal of the situation and their mental wellbeing, that appraisal of the COVID-19 pandemic as more threatening (i.e., high risk of getting infected and the infection being fatal, being overwhelmed with information and having difficulty controlling it, and being preoccupied with the pandemic situation) predicted higher anxiety. However, appraisal of the COVID-19 pandemic as more threatening did not predict lower curiosity. Both perceived risk and pandemic interest were weakly but positively related to curiosity. Thus, each of the three individual aspects of the pandemic situation appraisal may play a different role for anxiety and curiosity.

### 4.2 Personal resources appraisal and HE students wellbeing

Out of the two aspects of the resources' appraisal (secondary appraisal), both were related to anxiety and curiosity. Higher sense of control and self-efficacy predicted lower anxiety and higher curiosity. However, the relations between sense of control and self-efficacy and the outcome of anxiety and curiosity are to some extent opposite in nature. In a pandemic situation, sense of control, i.e., a feeling of having influence on what is happening, had the strongest association with anxiety as compared to other predictors. As mentioned above, the specificity of the early stage of the pandemic was not only its novelty but also the lack of reliable information and predictability. Thus, students high in dispositional sense of control, which is relatively independent of the current situation, could have controlled the level of anxiety more effectively. The question arises concerning how to implement sense of control in circumstances dominated by new, uncertain, changeable, and unpredictable events. This apparent contradiction can be explained by locus of control. A study among Norwegian and German-speaking participants showed that locus of control moderated the relation between COVID-19 stress and mental distress during the early months of the COVID-19 pandemic. An internal locus of control served as a buffer, whereas an external locus of control exacerbated this relation (46). People high in sense of control have a stronger need to feel connection between events in their life and their own actions. Thus, even in less controllable situations, they are more likely to find areas possible to control, like daily routine, thoughts, health behaviors or relationships, which may alleviate the level of anxiety. In the conditions of the pandemic, self-efficacy turned out to have a weaker association with anxiety but was the strongest predictor of curiosity. The curiosity-drive theories, which explain the nature of curiosity as a process to solve the challenge of lack of knowledge, indicate that people with stronger self-efficacy may be more motivated to face the challenges posed by lack of knowledge and thus be more willing to explore novel situations (47).

### 4.3. Health behaviors, social behaviors, contextual and demographic factors and HE students wellbeing

Another result of the study worth mentioning is that, similarly to the relations between sense of control and self-efficacy and the outcome of anxiety and curiosity, also the relations between preventive and health-promoting behaviors and anxiety and curiosity are to some extent opposite in nature. Higher levels of preventive behaviors were significantly related to higher anxiety, while higher levels of health-promoting behaviors were significantly related to higher curiosity. A predictive nature of these relations cannot be determined due to the cross-sectional design of this study. It is likely that higher anxiety triggers more preventive behaviors, such as self-isolation and high use of disinfection, while higher curiosity in the pandemic situation allows for more health-promoting behaviors, such as eating and sleeping healthy. A strong association between preventive behavior and anxiety during the COVID-19 pandemic has also been found in other studies (48, 49). However, the study by Wang et al. (50) showed an opposite finding, namely that greater adherence to preventive measures was linked to lower anxiety. The precise level of (high) anxiety may be of a key importance, with too high anxiety triggering avoidance and resistance. Cognitive processes such as the pandemic situation appraisal, e.g., pandemic interest and information stress related to information avoidance and unrealistic optimism, can play an important role in the anxiety–preventive behaviors relation. Other studies also indicate that people higher in trait curiosity generally tend to show more growth-oriented behaviors and have a greater sense of meaning in life (51). However, an opposite relation is also likely, with higher focus and performance of preventive behaviors resulting in an increase in anxiety and a higher level of health-promoting behaviors supporting state curiosity.

Social support is a broadly studied and widely accepted correlate of good mental and physical health. Previous studies indicate that college students with lower perceived social support were more likely to experience mental health problems (52). In contrast, in our study intensity of contacts with family, peers, neighbors, and lecturers had no significant relation with anxiety. However, the more intense students' contact with family and lecturers was, the higher their level of curiosity. This may indicate that these two sources of support might have been of a key importance for students' adaptive functioning expressed as curiosity in the unpredictable, and uncontrollable time of the pandemic. These results may also suggest that students with high curiosity were more willing to engage in academic work and stay in touch with their lecturers during the first wave of the pandemic, while for students with high COVID-19-related anxiety, studies and contact with lecturers was unimportant considering the global pandemic.

Hardly any contextual factors played a role in the prediction of anxiety and curiosity. There were only two exceptions. First, people who knew somebody who had been infected had significantly higher levels of anxiety compared to those who did not have this experience. Similar results were reported by Kregar Velikonja et al. (48). Second, students who perceived the current situation as more abnormal, e.g., with shortages of basic products and field hospitals being opened, had a higher level of curiosity. This relation showing that the more abnormal the situation was, the more willing to gain knowledge students become, seems adaptive. The insignificant relation between the COVID-19 situation index and anxiety is a bit surprising but as hypothesized, may likely be explained by the fact that the burden resulting from restrictions was similar for all students in this period of the pandemic. Coherently with previous studies (53, 54), female students had higher levels of anxiety (but not curiosity) than male students. This result indicates a need for additional female-focused psychological interventions in the HEI.

This study has several limitations. First, there is an overrepresentation of specific groups of students due to the online data collection method and the use of random snowball sampling. Ekman et al. (55) suggests that the bias associated with collecting information via online questionnaires is comparable to that of paper-and-pencil questionnaires. However, the fact that 74% of participants were women and 42% were students from medical universities might have influenced the levels of both outcome variables and selected predictors, particularly the appraisal of the pandemic situation, such as information stress. Additionally, there was a slight predominance of master's or 6-year medicine students compared to bachelor's degree students, suggesting that participants were more experienced students rather than novices. These factors highlight the need for caution when generalizing the study's findings. Second, self-developed indexes created for the purpose of this study were used instead of longer well-established psychological tests and scales, which may make the results more difficult to compare with other studies. This was, however, a conscious decision made to limit the length of the survey and make it more convenient for students who were already overwhelmed by the number of online activities. Finally, the cross-sectional nature of the study does not allow to draw definitive conclusions on the direction of predictions. However, this study included a large sample of HE students which provides unique information on students' mental functioning, allowing us to learn from that scenario for future challenges.

## 5 Conclusions and practical implications

The aim of the study was to investigate how primary appraisal (situation appraisal: information stress, perceived risk, pandemic interest) and secondary appraisal (appraisal of personal resources: self-efficacy and sense of control) predict students mental wellbeing (anxiety and curiosity). In challenging life situations such as the COVID-19 pandemic, the appraisal of personal resources seems to have the strongest association with students' mental wellbeing, with higher levels of sense of control and self-efficacy being associated with lower levels of anxiety and higher levels of curiosity. Pandemic situation appraisal had less impact on mental wellbeing than the appraisal of personal resources, with information stress having strongest association with anxiety.

Promoting students' personal coping resources is therefore crucial in order to strengthen their mental wellbeing in meeting future challenging situations in personal and professional life, both those experienced individually or concerning the general population (such as the recent pandemic). HE institutions should put more attention on creating a positive psychosocial and learning environment that supports the development of a wide range of personal resources, including self-efficacy and sense of control.

This calls not only for developing specific self-development courses but mostly requires a general change of a learning approach to more interactive and collaborative, as in opposition to strict or closed classroom situations, such an approach allows students to become more resistant, resilient and self-assured (56). With mental health being considered a great public health challenge, especially in this transitional phase of life, increasing awareness of the need for such changes among academic lecturers and HE institution authorities, seems crucial.

Our research clearly states how students subjective perspective matters for their mental wellbeing and that it should be included in mental-health promoting interventions. However, the intervention should be tailored to address other identified determinants of wellbeing, such as gender and health behaviors.

The COVID-19 pandemic is just an example of a highly demanding stressful situation. We cannot close our eyes to the upcoming threats with which future HE students will need to cope. The current pandemic can be treated as a lesson on how to support HE students to successfully navigate life's challenges and cultivate wellbeing.

## Data Availability

The raw data supporting the conclusions of this article will be made available by the authors, without undue reservation.
