# Supplementary material for: Mental wellbeing of higher education students in challenging times
Source: Front Public Health. 2025 Jan 7;12:1368443. doi: 10.3389/fpubh.2024.1368443 (PMC11746005; doi:10.3389/fpubh.2024.1368443)
Supplement: Supplementary file 1 [file Table_1.docx]

**Supplementary material S1a.** A Tools measuring Mental Well-being, Personal Resources Appraisal and COVID-19 Pandemic Situation’s Appraisal

Referring to the SARS-CoV-2 coronavirus pandemic and taking into account all areas of your life, to what extent over the past week ...

|  |  | 1 – not at all | 2 | 3 | 4 | 5 – very much so |
| --- | --- | --- | --- | --- | --- | --- |
| *Mental Well-being* | *Anxiety* |  |  |  |  |  |
|  | …have you been worried about what the next days will bring |  |  |  |  |  |
|  | …were you worried about what could happen |  |  |  |  |  |
|  |  |  |  |  |  |  |
|  | *Curiosity* |  |  |  |  |  |
|  | …were you full of enthusiasm |  |  |  |  |  |
|  | …did you feel lively and willing to act |  |  |  |  |  |
|  |  |  |  |  |  |  |
| *Appraisal of Personal Resources* | *Self-efficacy* |  |  |  |  |  |
|  | …were you confident that you could deal efficiently with unexpected events |  |  |  |  |  |
|  | …did you have a feeling that you could manage regardless of what happened to you |  |  |  |  |  |
|  |  |  |  |  |  |  |
|  | *Sense of control* |  |  |  |  |  |
|  | …have you felt unable to control the important things in your life |  |  |  |  |  |
|  | …have you had a feeling that you have no influence on what is happening around you |  |  |  |  |  |
|  |  |  |  |  |  |  |
| *COVID-19 Pandemic Situation Appraisal* | *Information stress* |  |  |  |  |  |
|  | …have you felt overwhelmed with information and have had difficulty controlling it |  |  |  |  |  |
|  | …have you experienced information chaos (conflicting, inconsistent information) that hindered your functioning |  |  |  |  |  |
|  | *Perceived own risk index* |  |  |  |  |  |
|  | How would you assess your risk of getting infected with SARS-CoV-2 coronavirus? | estimate value in % | | | | |
|  | How would you assess the risk that coronavirus infection can be fatal in your case? (estimate value in %) | estimate value in % | | | | |
|  | *Pandemic interest* |  |  |  |  |  |
|  | In general, how much are you interested in the coronavirus SARS-CoV-2 pandemic? | 1 – very little | 2 | 3 | 4 | 5 – very much |

Note. Results on the anxiety, curiosity, self-efficacy, sense of control and information scales are expressed as mean scores from the two given items, and higher scores indicated higher levels of the measured variables (with scores for sense of control being reversed). Perceived own risk index is calculated as a as a mean of estimations of the risk of getting infected with SARS-CoV-2 coronavirus and the risk of coronavirus infection being fatal.

**Supplementary material S1b.** COVID-19 Situation index

Which of the following apply to the country in which you live now?

| Due to COVID-19 pandemic: | Yes, at the moment | In the past but not anymore | Neither now not in the past |
| --- | --- | --- | --- |
| - we experience shortages of some products (e.g. toilet paper, soap, flour) |  |  |  |
| - everyone is recommended to stay at home |  |  |  |
| - all mass events are cancelled |  |  |  |
| - kindergartens and schools are closed |  |  |  |
| - universities are closed |  |  |  |
| - places where people often gather (e.g. restaurants, cinemas, malls) are closed |  |  |  |
| - there are reports of shortages in medical equipment |  |  |  |
| - borders are closed |  |  |  |
| - hospitals are overcrowded |  |  |  |
| - field hospitals are opened |  |  |  |
| - there are strict restrictions on leaving the house |  |  |  |
| - there is not enough space in the morgues for storing corpses of those who have died |  |  |  |

Note. The COVID-19 Situation Index is expressed as a mean score from the 12 items, with answers *neither now nor in the past* and *in the past but not anymore* = 0 and *yes, at the moment* = 1. A possible score of the index is between 0 and 1, with a higher score indicating more changes and limitations due to the COVID-19 pandemic.

**Supplementary Material S1c.** Preventive and Health-Promoting Behaviors Checklists

Below is a list of different behaviours. How often have you manifested them in the recent days (7-10 days)?

|  |  | 1 – not at all | 2 | 3 | 4 | 5 | 6 | 7 – all the time |  |
| --- | --- | --- | --- | --- | --- | --- | --- | --- | --- |
| Preventive Behaviors | - I stay at home whenever I can |  |  |  |  |  |  |  |  |
|  | - I meet with friends |  |  |  |  |  |  |  |  |
|  | - I avoid crowded places |  |  |  |  |  |  |  |  |
|  | - I shop as rarely as possible (max. once a week) |  |  |  |  |  |  |  |  |
|  | - I visit my relatives in old age |  |  |  |  |  |  |  |  |
|  | - I greet by shaking hands and / or embracing |  |  |  |  |  |  |  |  |
|  | - In the presence of other people, I keep at least a distance of 1.5-2 meters |  |  |  |  |  |  |  |  |
|  | - I wear gloves in public places |  |  |  |  |  |  |  |  |
|  | - I wear a mask in public places |  |  |  |  |  |  |  |  |
|  | - I disinfect objects (phone, car keys) and surfaces |  |  |  |  |  |  |  |  |
|  | - I disinfect shopping products or wash them well |  |  |  |  |  |  |  |  |
|  | - I wash my hands with soap for min. 30 seconds |  |  |  |  |  |  |  |  |
|  | - I disinfect my hands with an antibacterial gel. |  |  |  |  |  |  |  |  |
|  | - I am careful not to touch my eyes and mouth |  |  |  |  |  |  |  |  |
|  | - I cover my mouth with my elbow when I sneeze |  |  |  |  |  |  |  |  |
| Health-Promoting Behaviors | - I sleep at night for at least 7 hours |  |  |  |  |  |  |  | |
|  | - I drink 2 or more alcohol units per day (1 unit = 1 small beer / shot of vodka) |  |  |  |  |  |  |  | |
|  | - I exercise systematically (min. 30 min. at least 5 times a week) |  |  |  |  |  |  |  | |
|  | - I eat about 5 servings (400 g) of vegetables daily |  |  |  |  |  |  |  | |
|  | - I take vitamin D |  |  |  |  |  |  |  | |
|  | - I use relaxation techniques (yoga, meditation, mindfulness) |  |  |  |  |  |  |  | |

Note. The 15-item Preventive Behaviors and the 6-item Health-Promoting Behaviors Checklists’ scores were expressed as mean scores from the 15 and 6 items, respectively. Higher scores indicated higher levels of preventive and promoting behaviours.
