# Supplementary material for: Mental wellbeing of higher education students in challenging times
Source: Front Public Health. 2025 Jan 7;12:1368443. doi: 10.3389/fpubh.2024.1368443 (PMC11746005; doi:10.3389/fpubh.2024.1368443)
Supplement: Supplementary file 2 [file Table_2.docx]

**Supplementary Table 1.** Regression analysis of associations between demographic, contextual and social factors, health behaviours, Covid-19 situation appraisal, resources appraisal and student wellbeing (anxiety and curiosity) (n=3727).

|  |  |  | WELLBEING | | | | | | | | | | | | | | |  |
| --- | --- | --- | --- | --- | --- | --- | --- | --- | --- | --- | --- | --- | --- | --- | --- | --- | --- | --- |
|  |  |  | Anxiety | | | | | | |  | Curiosity | | | | | | | |
|  |  |  | B | SE B | BETA | R | Adjusted R^2^ | ΔR*^2^* | Sig. |  | B | SE B | BETA | R | Adjusted R^2^ | ΔR*^2^* | Sig. | |
| STEP 1  Demographic | (Constant) |  | 8.66 | 0.44 |  |  |  |  |  |  | 3.39 | 0.38 |  |  |  |  |  | |
|  | Age |  | 0.001 | 0.02 | 0.001 |  |  |  |  |  | 0.04 | 0.02 | .04** |  |  |  |  | |
|  | Gender |  | -1.29 | 0.09 | -0.24*** | .24 | .06 | .06 | <.001 |  | 0.28 | 0.08 | .06*** | .07 | .01 | .01 | <.001 | |
| STEP 2  Contextual | (Constant) |  | 9.18 | 0.48 |  |  |  |  |  |  | 2.66 | 0.42 |  |  |  |  |  | |
|  | Age |  | -0.01 | 0.02 | -0.01 |  |  |  |  |  | 0.05 | 0.02 | .05** |  |  |  |  | |
|  | Gender |  | -1.24 | 0.09 | -0.23*** |  |  |  |  |  | 0.28 | 0.08 | .06*** |  |  |  |  | |
|  | Covid-19 Situation Index |  | 0.60 | 0.22 | 0.05** |  |  |  |  |  | 0.60 | 0.19 | .06** |  |  |  |  | |
|  | Normal life |  | -0.30 | 0.04 | -0.13*** |  |  |  |  |  | 0.13 | 0.04 | .06*** |  |  |  |  | |
|  | Know Infected People |  | 0.38 | 0.10 | 0.06*** |  |  |  |  |  | 0.10 | 0.09 | .02 |  |  |  |  | |
|  | Quarantine |  | 0.03 | 0.18 | 0.002 | .29 | .08 | .03 | <.001 |  | -0.08 | 0.16 | -.01 | .10 | .01 | .01 | .001 | |
| STEP 3  Social | (Constant) |  | 8.99 | 0.52 |  |  |  |  |  |  | 0.93 | 0.45 |  |  |  |  |  | |
|  | Age |  | -0.01 | 0.02 | -0.01 |  |  |  |  |  | 0.05 | 0.02 | .05** |  |  |  |  | |
|  | Gender |  | -1.23 | 0.09 | -0.22*** |  |  |  |  |  | 0.35 | 0.08 | .07*** |  |  |  |  | |
|  | Covid-19 Situation Index |  | 0.62 | 0.22 | 0.05** |  |  |  |  |  | 0.66 | 0.19 | .06*** |  |  |  |  | |
|  | Normal life |  | -0.30 | 0.04 | -0.13*** |  |  |  |  |  | 0.12 | 0.04 | .06*** |  |  |  |  | |
|  | Know Infected People |  | 0.38 | 0.10 | 0.06*** |  |  |  |  |  | 0.05 | 0.09 | .01 |  |  |  |  | |
|  | Quarantine |  | 0.03 | 0.18 | 0.003 |  |  |  |  |  | -0.15 | 0.15 | -.02 |  |  |  |  | |
|  | Contact Family |  | -0.01 | 0.07 | -0.001 |  |  |  |  |  | 0.35 | 0.06 | .10*** |  |  |  |  | |
|  | Contact Peers |  | 0.000 | 0.04 | 0.000 |  |  |  |  |  | 0.13 | 0.04 | .06*** |  |  |  |  | |
|  | Contact Neighbours |  | -0.09 | 0.04 | -0.04* |  |  |  |  |  | 0.10 | 0.03 | .05** |  |  |  |  | |
|  | Contact Lecturers |  | 0.07 | 0.03 | 0.04** | .29 | .08 | .003 | .025 |  | 0.07 | 0.03 | .05** | .20 | .04 | .03 | <.001 | |
| STEP 4  Health behaviors | (Constant) |  | 7.86 | 0.54 |  |  |  |  |  |  | -0.39 | 0.47 |  |  |  |  |  | |
|  | Age |  | -0.01 | 0.02 | -0.01 |  |  |  |  |  | 0.04 | 0.02 | .04** |  |  |  |  | |
|  | Gender |  | -1.11 | 0.09 | -0.20*** |  |  |  |  |  | 0.45 | 0.07 | .10*** |  |  |  |  | |
|  | Covid-19 Situation Index |  | 0.29 | 0.22 | 0.02 |  |  |  |  |  | 0.38 | 0.19 | .04* |  |  |  |  | |
|  | Normal life |  | -0.26 | 0.04 | -0.11*** |  |  |  |  |  | 0.11 | 0.03 | .06*** |  |  |  |  | |
|  | Know Infected People |  | 0.35 | 0.10 | 0.05*** |  |  |  |  |  | 0.05 | 0.09 | .01 |  |  |  |  | |
|  | Quarantine |  | 0.01 | 0.17 | 0.001 |  |  |  |  |  | -0.11 | 0.15 | -.01 |  |  |  |  | |
|  | Contact Family |  | 0.02 | 0.07 | 0.004 |  |  |  |  |  | 0.25 | 0.06 | .07*** |  |  |  |  | |
|  | Contact Peers |  | 0.01 | 0.04 | 0.01 |  |  |  |  |  | 0.10 | 0.04 | .05** |  |  |  |  | |
|  | Contact Neighbours |  | -0.05 | 0.04 | -0.02 |  |  |  |  |  | 0.09 | 0.03 | .05** |  |  |  |  | |
|  | Contact Lecturers |  | 0.05 | 0.03 | 0.03 |  |  |  |  |  | 0.07 | 0.03 | .05** |  |  |  |  | |
|  | HB_Prev |  | 0.45 | 0.04 | 0.20*** |  |  |  |  |  | -0.06 | 0.03 | -.03 |  |  |  |  | |
|  | HB_Promo |  | -0.32 | 0.03 | -0.15*** | .364 | .13 | .05 | <.001 |  | 0.51 | 0.03 | .28*** | .33 | .11 | .07 | <.001 | |
| STEP 5  Pandemic situation appraisal (primary appraisal) | (Constant) |  | 2.51 | 0.43 |  |  |  |  |  |  | 1.18 | 0.47 |  |  |  |  |  | |
|  | Age |  | 0.03 | 0.01 | 0.02 |  |  |  |  |  | 0.03 | 0.02 | .03 |  |  |  |  | |
|  | Gender |  | -0.58 | 0.07 | -0.11*** |  |  |  |  |  | 0.28 | 0.07 | .06*** |  |  |  |  | |
|  | Covid-19 Situation Index |  | 0.17 | 0.17 | 0.01 |  |  |  |  |  | 0.45 | 0.18 | .04** |  |  |  |  | |
|  | Normal life |  | -0.13 | 0.03 | -0.05*** |  |  |  |  |  | 0.08 | 0.03 | .04* |  |  |  |  | |
|  | Know Infected People |  | 0.19 | 0.08 | 0.03** |  |  |  |  |  | 0.07 | 0.08 | .01 |  |  |  |  | |
|  | Quarantine |  | -0.10 | 0.13 | -0.01 |  |  |  |  |  | -0.10 | 0.14 | -.01 |  |  |  |  | |
|  | Contact Family |  | -0.07 | 0.05 | -0.02 |  |  |  |  |  | 0.27 | 0.06 | .08*** |  |  |  |  | |
|  | Contact Peers |  | -0.08 | 0.03 | -0.03* |  |  |  |  |  | 0.13 | 0.03 | .06*** |  |  |  |  | |
|  | Contact Neighbours |  | -0.07 | 0.03 | -.03** |  |  |  |  |  | 0.09 | 0.03 | .05** |  |  |  |  | |
|  | Contact Lecturers |  | 0.01 | 0.02 | 0.003 |  |  |  |  |  | 0.09 | 0.02 | .05*** |  |  |  |  | |
|  | HB_Prev |  | 0.19 | 0.03 | 0.08*** |  |  |  |  |  | -0.04 | 0.03 | -.02 |  |  |  |  | |
|  | HB_Promo |  | -0.13 | 0.03 | -0.06*** |  |  |  |  |  | 0.45 | 0.03 | .25*** |  |  |  |  | |
|  | Perceived own risk |  | 0.01 | 0.002 | 0.06*** |  |  |  |  |  | 0.000 | 0.002 | .000 |  |  |  |  | |
|  | Information stress |  | 0.55 | 0.01 | 0.59*** |  |  |  |  |  | -0.18 | 0.01 | -.23*** |  |  |  |  | |
|  | Pandemic interest |  | 0.30 | 0.03 | 0.12*** | .70 | .49 | .36 | .001 |  | 0.07 | 0.03 | .03 | .40 | .16 | .05 | <.001 | |
| STEP 6  Resources appraisal (secondary appraisal) | (Constant) |  | 5.82 | 0.39 |  |  |  |  |  |  | -1.05 | 0.42 |  |  |  |  |  | |
|  | Age |  | 0.03 | 0.01 | 0.02* |  |  |  |  |  | 0.01 | 0.01 | .01 |  |  |  |  | |
|  | Gender |  | -0.20 | 0.06 | -0.04*** |  |  |  |  |  | -0.11 | 0.07 | -.02 |  |  |  |  | |
|  | Covid-19 Situation Index |  | 0.003 | 0.15 | 0.000 |  |  |  |  |  | 0.44 | 0.16 | .04** |  |  |  |  | |
|  | Normal life |  | -0.05 | 0.03 | -0.02 |  |  |  |  |  | 0.02 | 0.03 | .01 |  |  |  |  | |
|  | Know Infected People |  | 0.16 | 0.07 | 0.03* |  |  |  |  |  | 0.04 | 0.07 | .01 |  |  |  |  | |
|  | Quarantine |  | -0.18 | 0.12 | -0.02 |  |  |  |  |  | -0.03 | 0.13 | -.003 |  |  |  |  | |
|  | Contact Family |  | 0.06 | 0.05 | 0.02 |  |  |  |  |  | 0.13 | 0.05 | .04** |  |  |  |  | |
|  | Contact Peers |  | 0.02 | 0.03 | 0.01 |  |  |  |  |  | 0.02 | 0.03 | .01 |  |  |  |  | |
|  | Contact Neighbours |  | -0.02 | 0.03 | -0.01 |  |  |  |  |  | 0.04 | 0.03 | .02 |  |  |  |  | |
|  | Contact Lecturers |  | 0.03 | 0.02 | 0.01 |  |  |  |  |  | 0.06 | 0.02 | .04** |  |  |  |  | |
|  | HB_Prev |  | 0.18 | 0.03 | 0.08*** |  |  |  |  |  | -0.003 | 0.03 | -.001 |  |  |  |  | |
|  | HB_Promo |  | -0.01 | 0.03 | -0.003 |  |  |  |  |  | 0.28 | 0.03 | .15*** |  |  |  |  | |
|  | Perceived own risk |  | 0.01 | 0.001 | 0.04*** |  |  |  |  |  | 0.004 | 0.002 | .03* |  |  |  |  | |
|  | Information stress |  | 0.23 | 0.01 | 0.25*** |  |  |  |  |  | 0.02 | 0.02 | .03 |  |  |  |  | |
|  | Pandemic interest |  | 0.25 | 0.03 | 0.10*** |  |  |  |  |  | 0.09 | 0.03 | .04** |  |  |  |  | |
|  | Self efficacy |  | -0.11 | 0.01 | -0.11*** |  |  |  |  |  | 0.40 | 0.01 | .44*** |  |  |  |  | |
|  | Sense of control |  | -0.43 | 0.02 | -0.46*** | .79 | .61 | .12 | <.001 |  | 0.15 | 0.02 | .18*** | .60 | .36 | .20 | <.001 | |

*Note*. Dichotomous variables were coded as follows: - *gender*: female = 0, male = 1; *know infected people*: no=0, yes =1; *been* *quarantined*: never = 0, at least once = 1; *providing help*: never = 0, at least once = 1.
HB_Prev – Preventive health behaviours; HB_Promo – health promoting behaviours.

The entire group of predictors significantly predicted anxiety F(17, 3709) = 350.1, *p* < .001, adjusted R*^2^* = .61 and curiosity F(17, 3709) = 122.1, *p* < .001, adjusted R*^2^* = .36.

* *p* < .05, ** *p* < .01, *** *p* < .001.
